# Natural resource-derived NiO nanoparticles via aloe vera for high-performance symmetric supercapacitor

**DOI:** 10.1038/s41598-024-57606-w

**Published:** 2024-03-28

**Authors:** Mamta Bulla, Vinay Kumar, Raman Devi, Sunil Kumar, Avnish Kumar Sisodiya, Rita Dahiya, Ajay Kumar Mishra

**Affiliations:** 1 Department of Physics, COBS&H, CCS Haryana Agricultural University, Hisar, Haryana 125004 India; 2 Department of Physics, Ramjas College, University of Delhi, Delhi, 110007 India; 3 Department of Chemistry, Durban University of Technology, Steve Biko Road, Durban, 4001 South Africa

**Keywords:** Green approach, Aloe vera, Nickel oxide, Nanoparticles, Symmetric supercapacitor, Materials science, Physics

## Abstract

This investigation reported a one-step green synthesis of nickel oxide nanoparticles (NiO NPs) using aloe vera leaves extract solution for their application in a supercapacitor. This method used aloe vera leaves as a reducing agent, which is very simple and cost-effective. The synthesized NPs were thoroughly characterized using various techniques. The X-ray diffraction analysis unequivocally confirmed the crystalline nature; field emission scanning electron microscopy and transmission electron microscopy images showed different shapes and forms of an agglomerated cluster of synthesized NPs. The absorption spectra were recorded from UV visible spectroscopy, while Fourier transform infrared spectroscopy provided insights into the functional groups present. Electrochemical assessments were carried out via cyclic voltammetry, galvanostatic charging-discharging and electrochemical impedance spectroscopy. These experiments were performed using a 2 M KOH electrolyte within a 1.0 V potential window. Impressively, the single electrode displayed a remarkable specific capacitance of 462 F g^−1^ at a scan rate of 1 mV s^−1^ and 336 F g^−1^ at a current density of 0.76 A g^−1^. Further, a symmetric two-electrode device (NiO||NiO) has been successfully fabricated by employing a separator between the electrodes. The device exhibited an exceptional specific capacitance of approximately 239 F g^−1^, along with an energy density of 47.8 Wh kg^−1^ and a power density of 545 W kg^−1^ at 1 A g^−1^ current density within a 1.2 V potential window. The fabricated device also shows a retention capacity of 89% at 10 A g^−1^ after 2000 cycles with 114% of columbic efficiency. The present study underscores the effectiveness of the green synthesis approach in producing NiO NPs and establishes their potential as highly promising candidates for supercapacitor applications, showcasing both excellent electrochemical performance in a three-electrode system and remarkable stability in a practical two-electrode device. The results collectively highlight the efficacy of the green approach in producing NiO NPs, establishing its potential as a highly promising candidate for supercapacitor application.

## Introduction

The advancement of electronics has sparked greater attention towards energy storage systems. Besides this, the lessening of fossil fuels and their hazardous consequence on the ecosystem and human health led to the evolution of energy storage systems from renewable sources^1,2^. Supercapacitors (SCs), fuel cells and second-generation lithium-ion batteries are considered highly promising options for energy storage devices because of their high energy density, power density, long cyclability, high sustainability and environment-friendly nature^3,4^. Specifically, SCs and batteries are the primary energy storage devices and developing devices to store energy effectively from renewable resources is a major concern^5^. SCs are gaining much attention because they have a higher specific power, fast charging-discharging and prolonged cyclability than batteries, which are used in different fields including electric vehicles, portable electronic devices, automobiles, etc.^6^. According to the basic principles of charge storage systems, SCs can be classified into two categories: electric double-layer capacitors (EDLC) and pseudo capacitors^7^. In EDLC, the charge will be stored electrostatically, including carbonaceous materials. Besides this, pseudo-capacitors store the charge faradically and employ transition metal oxide (RuO_2_, ZnO, NiO and MnO_2,_ etc.)^8^, metal sulphides^9^ and conducting polymers such as polyaniline (PANI) as an electrode material^10,11^. These materials allow a fast, reversible faradaic redox reaction and provide specific capacitance 10–100 times higher than EDLC^12^. Therefore, pseudo-capacitor development has become a thrust area to produce high specific capacitance and power density. Especially transition metal oxide NPs catch extensive biological and non-biological applications because of their physical and chemical properties, different morphology, shape, size, different crystalline phases, high surface-to-volume ratio, electric, magnetic and optical properties^13^. Among other metal oxides (RuO_2_, ZnO, NiO, MnO_2_, etc.), NiO has gained major attention because of its tunable band gap, high thermo-chemical stability, low toxicity, lesser cost and low-cost environmental impact and excellent pseudocapacitive behaviour^8,14^. With very few amounts of Ni^+3^ and Ni^+2^ ions in the crystal lattice, NiO is a p-type semiconductor in which holes carry the majority of the current^15^. It also shows substantial electrochemical, magnetic, antioxidant and catalytic properties^16^. Moreover, NiO nanoparticles may be employed as a dye-sensitized photocathode, a lithium-ion battery anode, or an SCs electrode in electrochemical systems and catalytic processes.

Various processes are used to synthesize the nanomaterials, such as hydrothermal, co-precipitation, sol–gel, etc.^17^. However, these approaches are pretty expensive and involve toxic chemicals which have a detrimental impact on the environment. Therefore, the urge has been shifted to a green approach for synthesizing NPs. Green and phyto-synthesis are the processes for synthesizing nano-sized metal oxide using plant extract, enzymes and micro-organisms^18^. The green approach of NPs is gaining attention from researchers and scientists owing to its cost-effectiveness, rapidity, safety and environmental-friendliness. Plant extracts have taken much consideration out of these different biomaterials due to their availability, effectiveness and green features. Plant extracts contain various potent antioxidants, including amino acids, polyphenols, nitrogenous bases and reducing sugars. These act as a capping agent through an organizational action by trapping the metal ions in the amylose helix at specific locations and the surface of plant leaves acts as a bio template that controls the NP size and prevents the agglomeration of particles^19^. For instance, Nwanya et al., 2020 reported the water-mediated synthesis of NiO from *Zea mays* lea silk extract and found a specific capacitance of 54 F g^−1^ at 5 mV s^−1^^20^. Moreover, Reddy et al., 2019 fabricated NiO NPs using *Moringa oleifera* plant extract through co-precipitation and found that synthesized NPs display a specific capacitance of about 350 F g^−1^ at a scan rate of 0.5 mV s^−1^^21^. Aloe vera is a succulent plant of the Liliaceae family that has been used for centuries in traditional medicine for its numerous health benefits and cultivated around 23,600 hectares worldwide^22^. Also, it can be cultivated in hot and dry climates (with less water), like a cactus plant^23^. Aloe vera is a plant that is high in water content and rich in polysaccharides, vitamins, saponins, polyphenols, etc. This has emerged as a viable alternative to synthetic surfactants in nano synthesis and can function as a natural surfactant, solvent, bio-template and stabilizing agent for synthesizing nanometal oxides^24^. Recently, aloe vera plant extract from an aqueous solution of metal ions was used to generate gold and silver NPs^25^.

This investigation reported a single-step green approach for synthesizing NiO NPs via a solution derived from aloe vera leaves for supercapacitor application. The aloe vera leaves were used as a reducing agent. The structural, optical and morphological characteristics of this synthesized material have been investigated using an array of techniques, including X-ray diffraction (XRD), Fourier transforms infrared (FTIR), UV–visible spectroscopy, Field emission scanning electron microscopy (FE-SEM) and Transmission electron microscopy (TEM). The electrochemical behaviour of the material was investigated using cyclic voltammetry (CV), galvanostatic charging-discharging (GCD) and electrochemical impedance spectroscopy (EIS). The novelty of this work is the fabrication of an SCs device from aloe vera plant extract that provides a high pseudo-capacitance and stability due to high-density faradaic activities.

## Results and discussion

### X-ray diffraction

**Figure 1:**
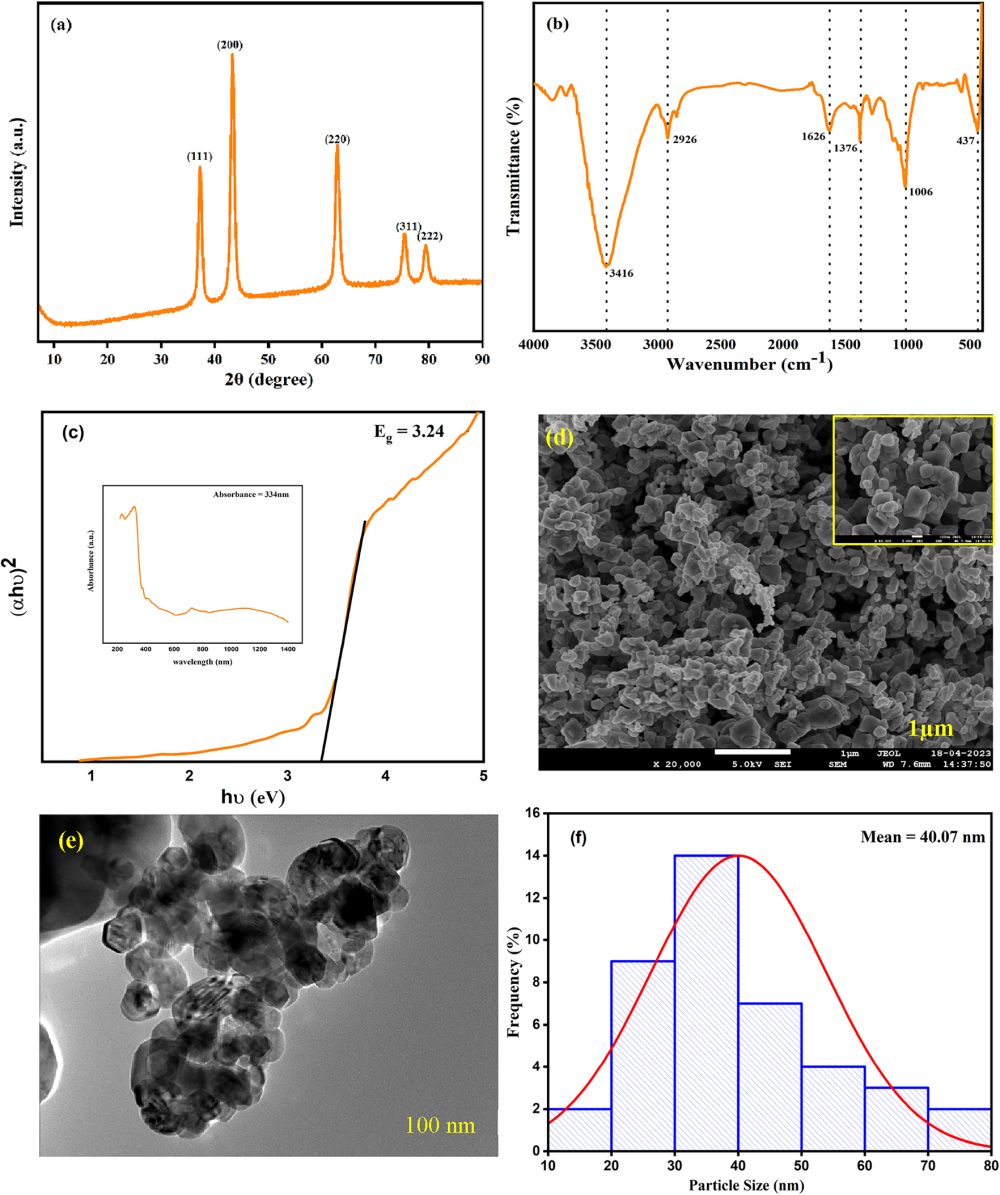
Represents (**a**) XRD (**b**) FT-IR. (**c**) UV Spectra, (**d**) FE-SEM image (**e**) TEM image, (**f**) Histogram of diameter distribution.

The XRD pattern of synthesized NiO NPs is depicted in Fig. 1a, revealing the five most prominent diffraction peaks which are observed at 37.3°, 43.3°, 62.8°, 75.6° and 79.4° of 2θ values. These peaks are indexed by the crystal planes (111), (200), (220), (311) and (222), respectively and correspond to the face-centred cubic (FCC) structure arrangement of NPs that confirm the production of material with high purity (as indicated by JCPDS card No. 47-1049)^26^. Additionally, the mean of sin^2^θ of the first two peaks provided the confirmation of FCC structure, which was found to be 0.78^27^. The NiO NPs displayed an average crystallite size of 16 nm, derived from calculations using the Scherrer formula provided below^28^:

1 $$D=\frac{0.9\lambda }{\beta cos\theta }$$

where $\lambda$ stands for X-ray wavelength, β is the full-width half maxima and $\theta$ represents Bragg’s angle, respectively.

### FT-IR

Figure 1b illustrates the FT-IR spectra of synthesized NiO NPs. The observed peak at 437 cm^−1^ is due to the stretching vibration of nickel and oxygen. Surface hydration can be seen by a broad peak at 3416 cm^−1^ and a tiny peak at 1626 cm^−1^. The presence of C–H stretching is evident through a peak at 2926 cm^−1^. Furthermore, peaks at 1376 cm^−1^ and 1006 cm^−1^ provide confirmation of the presence of C=O and C–O stretching, indicating the existence of aromatic rings and polyphenols^29^.

### UV-spectroscopy

The absorption spectra for NiO are shown in Fig. 1c with a range of 200–800 nm. The absorption peak was observed at 334 nm because of inter-band π–π* electronic transition. The direct energy band gap was determined using Tauc’s plot Eq. (2) and found to be 3.24 eV^30^:

2 $${\left(\alpha h\nu \right)}^{n}=A\left(h\nu -{E}_{g}\right)$$

where $\alpha$ stands for the absorption coefficient, A represents absorbance, hν denotes photon energy, E_g_ signifies energy band gap and n characterizes the nature of the transition process.

### FE-SEM

The two-dimensional high magnification structure of synthesized NiO NPs at different scales was examined using FE-SEM as presented in Fig. 1d. The resulting images revealed diverse shapes in the NiO NPs, which aggregated to form clusters. These observations indicate a high degree of agglomeration, resulting in clusters comprising individual nanoparticles. The presence of larger nanoparticles can be attributed to the inherent tendency of NiO nanoparticles to agglomerate, driven by their elevated surface energy and ultrafine nature, leading to high surface tension. Furthermore, the irregular shapes and sizes of the NiO NPs stem from the non-uniform nucleation process^17^.

### TEM

Transmission Electron Microscopy (TEM) stands as a potent analytical technique utilized for the visualization and characterization of nanoparticles, enabling the precise assessment of their geometric attributes such as shape, dimensions and structural characteristics. In Fig. 1e, TEM images of synthesized NiO nanoparticles are depicted at a scale of 100 nm. The analysis of these images corroborates the heterogeneous nature of the nanoparticles, showcasing diversity in both shape and size, a feature consistent with observations made through FESEM. Exploiting ImageJ software, the average nanoparticle size was calculated by computing the mean size of all particles, as illustrated in the histogram represented in Fig. 1f, which found a value of 40.07 nm. Especially the images reveal the presence of individual particles as well as aggregated formations, contributing to the comprehensive understanding of the nanoparticle morphology^31^.

### Electrochemical study for single electrode

**Figure 2:**
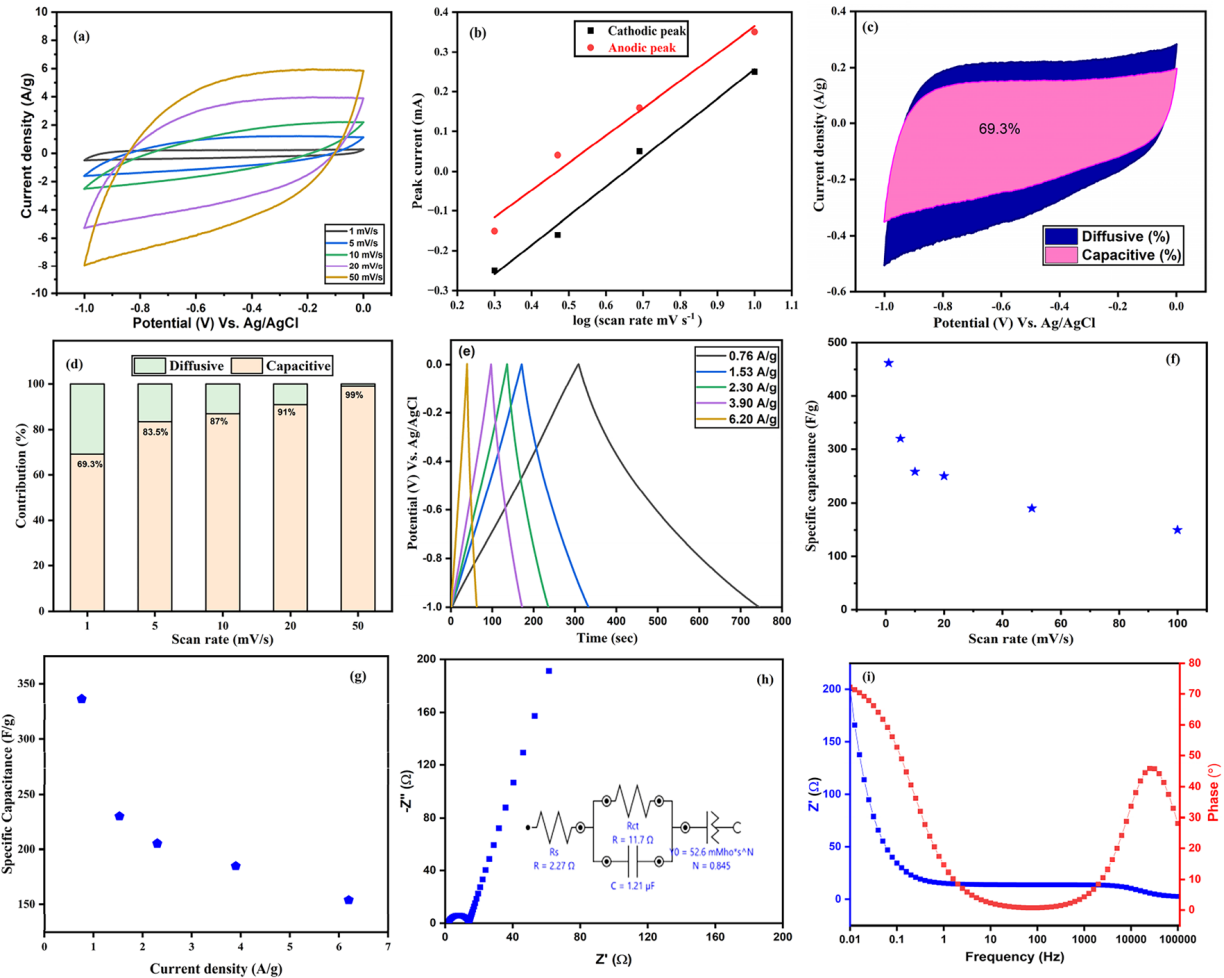
Represents (**a**) CV plot at different scan rates, (**b**) linear fitting of log (peak current) versus log (scan rate), (**c**) CV curves at the 5 mV/s, (**d**) capacitive/diffusive contribution at several scan rates, (**e**) GCD plot at different current densities, (**f**) specific capacitance at different scan rates, (**g**) specific capacitance at different current densities, (**h**) Nyquist plot and (**i**) Bode plot.

Figure 2a represents the cyclic voltammograms for the working single electrode at several scan rates such as 1, 5, 10, 20 and 50 mV s^−1^ in a voltage window − 1.0 to 0.0 V (detailed information of optimized voltage window in “Supplementary File”). The area enclosed within the CV curve represents the total stored charge. The specific capacitance for different scan rates was calculated using Eq. (8) and found to be 462, 432, 320, 250 and 190 F g^−1^ at 1, 5, 10, 20 and 50 mV s^−1^ respectively. The broad anode and cathode peaks are because of the faradic redox reaction occurring on the electrode active surface. As the redox reactions are kinetically irreversible, the reduction and oxidation peaks are not symmetric at cathodic and anodic scans, respectively. The interchange of electrons and ions has brought this kinetic irreversible nature of the redox process. During the cathodic scan (− 1.0 V to 0.0), the reduction of Ni^3+^ to Ni^2+^ is due to ions and electron intercalation in 2 M aqueous KOH. External fields prompt electron and ion movement through the electrolyte so that the electrons recombine with surface ions, diffusing into the active material, resulting in a reaction near the NiO electrode surface. Furthermore, the de-intercalation of electrons and ions during an anodic scan (0.0 to − 1.0 V) originates the oxidation of Ni^2+^ to Ni^3+^. This highlights the significant redox process variation caused by intercalation/de-intercalation in the working electrode^32^. The mechanism involved in the redox reaction can be explained by the equation^33^, which is given below:

3 $${i}_{p}=a{\nu }^{b}$$

4 $$\mathit{log}{i}_{p}=b log\nu +loga$$

where i_p_ is the peak current, ν is the scan rate, a and b are constants.

It was observed that as scan rates increase, current peak also increases and shifts to cathodic potential towards cathodic voltage and in the same manner shift on anodic voltage towards the anodic side shown in Fig. 2b. This increase in peak current shows the super-capacitive nature of the electrode. According to Eq. (3), peak current both anodic and cathodic, is directly proportional to the ν^b^. The geometric fitting of Eq. (4) is depicted in Fig. 2b and the intercept and slope are used to estimate the values of constants a and b, respectively. The diffusion-controlled process takes over when b is close to 0.5 and the capacitive-controlled mechanism operates when b is close to 1. From the slope of Eq. (4), b is calculated as 0.68 indicating both diffusive and capacitive mechanism controlling the reaction in 2 M KOH electrolyte^34^. Beside this, diffusive and capacitive contribution can be calculated using Dunn’s equation which is given below^35^:

5 $${i}_{p}={k}_{1}\nu + {k}_{2}{\nu }^\frac{1}{2}$$

6 $$\frac{{i_{p} }}{{\nu^{{{\raise0.7ex\hbox{$1$} \!\mathord{\left/ {\vphantom {1 2}}\right.\kern-0pt} \!\lower0.7ex\hbox{$2$}}}} }} = k_{1} \nu^{{{\raise0.7ex\hbox{$1$} \!\mathord{\left/ {\vphantom {1 2}}\right.\kern-0pt} \!\lower0.7ex\hbox{$2$}} + }} k_{2 }$$

Here k_1_ν and k_2_ ν^1/2^ are the factor representing the capacitive current due to surface redox reaction and diffusive current contribution due to ion intercalation-deintercalation, respectively. The value of k_1_ and k_2_ can be obtained by fitting Eq. (6). The capacitive and diffusive response with their respective contribution at various scan rate is depicted in Fig. 2d. It is clearly seen diffusion contribution is decreasing because ions do not get enough time for intercalation-deintercalation inside the working electrode. This can be explained by the fact that electro-active materials are less available at high scan rates in comparison to low ones. At lower scan rates, both the inner and outer surfaces of the electrode are involved in charge storage, yet at higher scan rates, only the surface region of the working electrode is accessible and ionic diffusion takes place only. Also, Fig. 2f shows that specific capacitance decreased with increasing scan rates. The minimum capacitive contribution was 69.3% at lower scan rate of 1 mV s^−1^ (Fig. 2c) and maximum contribution of 99% reached at higher scan rate of 50 mV s^−1^. However, at the higher scan rates, the effect of the inner materials is reduced due to the deficiency in the internal porosity of the synthesized material^36^.

The GCD was performed at the various current densities i.e., 0.76, 1.53, 2.3, 3.9 and 6.2 A g^−1^ of working electrode. The de-formed triangular shape for all GCD plots confirms the pseudo-capacitive behaviour of the material (Fig. 2e). The specific capacitance was determined for different current densities from Eq. (9) and found to be 336 F g^−1^ at 0.76 A g^−1^ and 153.84 F g^−1^ at 6.2 A g^−1^. Due to the insufficient availability of the whole electrode at higher current densities, the specific capacitance decreased with increasing current density shown in Fig. 2g ^37^.

EIS was further studied to check the electrochemical behaviour of the working electrode correlated with the electrode resistance. Both electronic and ionic species have an impact on the electrochemical impedance. The electronic component is due to inherent electrical resistance existing between the material and the substrate and the ionic from the resistances related to both the electrolyte and the diffusion of ions through electrode pores^20^. EIS was conducted at 0.5 V (vs. Ag/AgCl), spanning frequencies from 0.01 to 10^5^ Hz. The Nyquist plot for NiO, displayed in Fig. 2h, was generated using ZSimpWin software, incorporating an embedded equivalent circuit. The impedance data were then fitted using the circuit values. The plot displays a semi-circle at a higher frequency and near a straight line (greater than 45°-angled) at a lower frequency. The solution resistance (R_s_ = 2.27 Ω) corresponds to the electrolyte solution, the charge transfers resistance (R_ct_ = 11.7 Ω) that occurs at the interface of electrode–electrolyte and the constant phase element (CPE) that is present due to the electrode–electrolyte surface can all be seen in the circuit. The intercalation of ions on the NiO electrode in the plot can be separated into three mechanism phases. The semi-circle at high frequency displays the movement of ions at the interface of the electrolyte–electrode. The second middle-frequency region illustrates the charge-transfer mechanism throughout the reaction and the diameter of the semi-circle corresponds to R_ct_. The last lower frequency region (straight line) is analogous to the diffusion of ions into the active material^38^. Therefore, the electrical conductivity and surface area of the working electrode are associated with charge-transfer resistance, while the straight line symbolizes the diffusion of electroactive ions.

The bode plot of impedance magnitude |z| and phase angle (°) with the frequency function is displayed in Fig. 2i. The bode plot illustrates that at lower frequencies there is an increase in impedance magnitude and a decrease in impedance magnitude near the higher frequencies. The area at which impedance is not dependent on frequency provides the storage ability of the active electrode although the value of |z| gives the value R_s_ where the impedance is almost independent of frequency^39^. The phase angle of the working electrode was about 70°, which confirms that it can be used for practical application.

### Electrochemical analysis for the device

**Figure 3:**
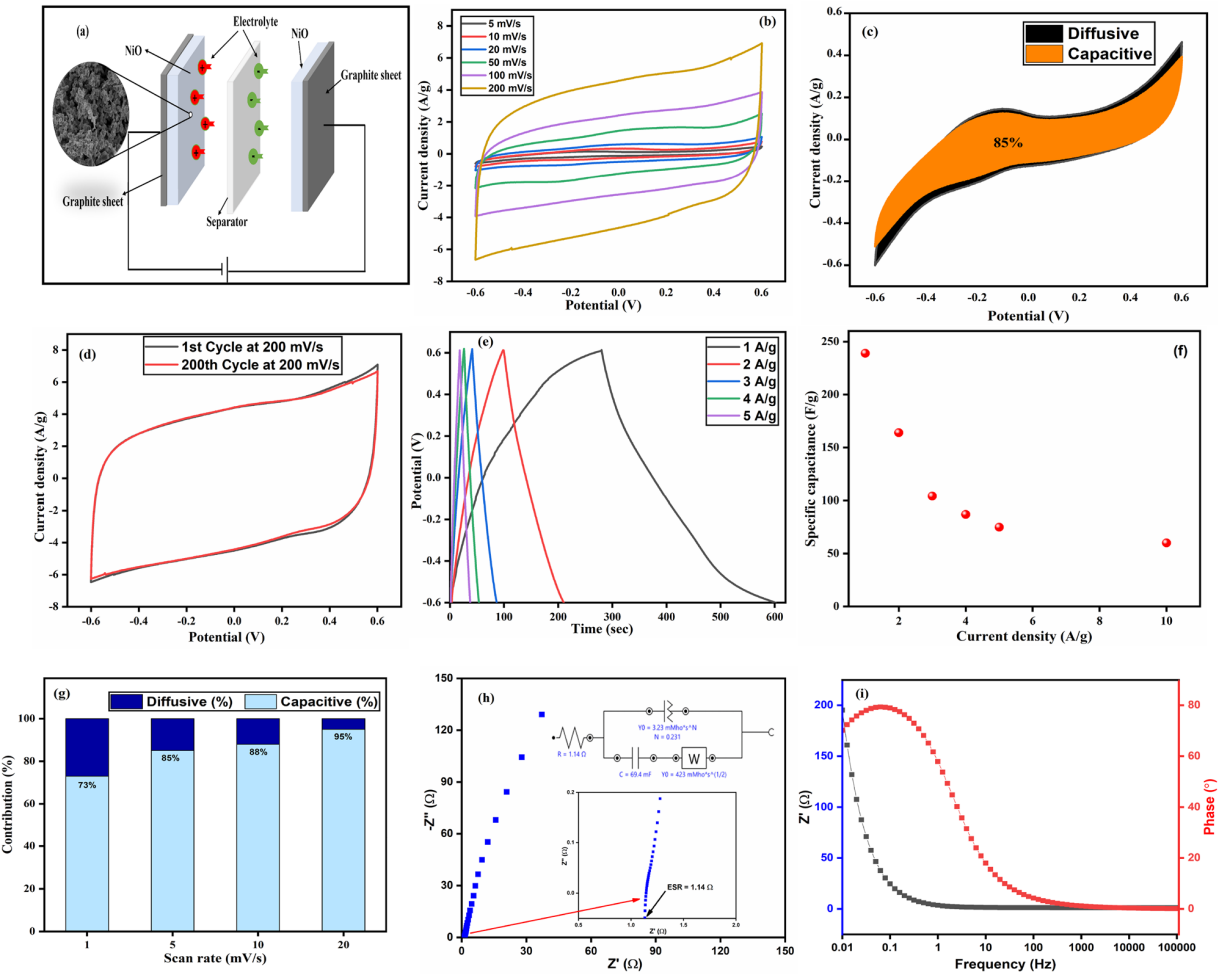
Represents (**a**) fabricated device, (**b**) CV plot at different scan rate, (**c**) CV curves at the 5 mV/s, (**d**) CV curve after 200 cycles, (**e**) GCD plot at different current densities, (**f**) specific capacitance at different current densities, (**g**) capacitive/diffusive contribution at several scan rates, (**h**) Nyquist plot and (**i**) Bode plot.

The fabrication of a two-electrode symmetric SC device (Fig. 3a) using synthesized material is described in the experimental section. Similar masses (1.2 mg) were loaded on both the cathode and anode electrodes. The CV was performed in a 1.2 V potential window for the device at various scan rates from 5 to 200 mV s^−1^ as shown in Fig. 3b. The CV curve shape is maintained even at higher scan rates showing the high-rate ability of the symmetric device. Also, Fig. 3d presented that at 200 mV s^−1^ scan rate, the curve is almost maintained after 200 cycles, which shows the stability of the device. At a 5 mV s^−1^ scan rate, the greater amount of electrolyte ion interaction provided a specific capacitance of 180 F g^−1^. With increasing scan rate, diffusive behavior reduces due to insufficient time for intercalation of ions into electrode as shown in Fig. 3g. The diffusive-capacitive behavior was studied as discussed earlier section (Eq. 6); at a lower scan rate of 1 mV s^−1^ the capacitive behavior was found to be 73% (Fig. 3c) and the maximum was 95% at a higher scan rate of 20 mV s^−1^.

**Figure 4:**
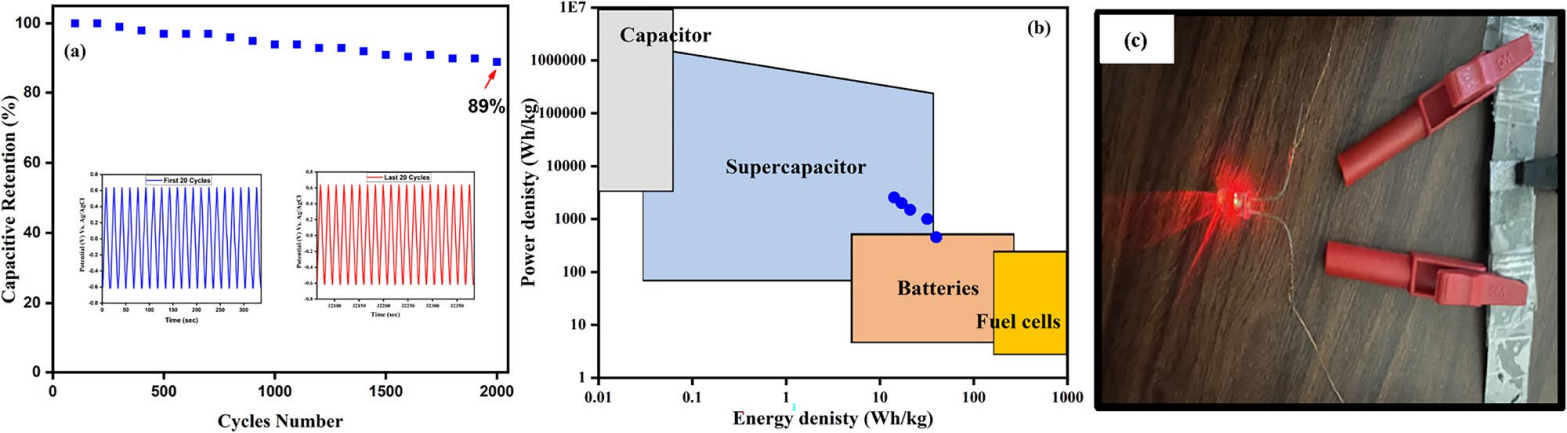
Represents (**a**) 2000 Cycles of charging-discharging, (**b**) Ragone plot and (**c**) Red LED is enlightened by two symmetric devices coupled in series.

The GCD plot at various current densities varying from 1 to 5 A g^−1^ within a 1.2 V voltage window is depicted in Fig. 3e. The non-linear shape of the GCD plot for the device owing to the pseudo-capacitive behaviour. The voltage drop is related to an internal resistance that is found negligible in the GCD plot. The performance was evaluated using the GCD plot of the symmetric device and further calculated the specific capacitance at all current densities. A capacitance of 239 F g^−1^ was observed at a current density of 1 A g^−1^ and 56 F g^−1^ at 10 A g^−1^ so with an increase in current density, the specific capacitance decreased (Fig. 3f). The energy density and power density for a single electrode and device at different current densities are summarized in Table 1. The energy density and power density at 1 A g^−1^ were observed 39.8 Wh kg^−1^ and 453.7 W kg^−1^, which lies between the SCs and batteries, as displayed in the Ragone plot (Fig. 4b). This device showed an enhanced energy density and power density as compared to the previously reported literature, asymmetric device of NiO//AC showed energy density of 52.4 Wh kg^−1^ at a power density of 800 W kg^−1^^40^, hybrid two-dimensional nickel oxide-reduced graphene oxide nanosheets showed 5.4 Wh kg^−1^ energy density at an of 0.43 kW kg^−1^ power density^41^, NiO single electrode showed 3.3 kW kg^−1^ and 27 Wh kg^−1^^42^ and NiWO_4_//AC showed 15.1 Wh kg^−1^energy density at 4.8 kW kg^−1^ power density^43^.

**Table 1:** Summary of energy density and power density for single electrode and device.

Measurements	Single Electrode					Device				
Current density (A g^−1^)	0.7	1.53	2.3	3.9	6.2	1	2	3	4	5
Energy density (Wh kg^−1^)	46.6	31.9	28.4	25.6	21.4	39.8	32	21	17	14.2
Power density (W kg^−1^)	379.5	764	1150	1951.4	3076.8	453.7	1001.5	1500	2000	2556

For practical everyday applications, the stability of the device is crucial. Therefore, to check the stability of the device GCD was performed at a fixed current density of 10 A g^−1^ for 2000 cycles and found 89% of capacity retention as presented in Fig. 4a. Also, after 2000 cycles of charging-discharging, the device maintained 114% of columbic efficiency. The red LED was successfully illuminated for the practical demonstration by combining two symmetric devices in series, as illustrated in Fig. 4c.

The EIS is essential for the electrochemical behaviour of the device and is performed at 0.5 V with frequencies varying from 0.1 to 10^5^ Hz. The results from EIS are shown through the Nyquist and Bode plot as depicted in Fig. 3h,i. Using ZSimpWin software, a Nyquist plot was generated for the device, featuring an embedded equivalent circuit, as illustrated in Fig. 3g. The absence or minuteness of a semicircle in the plot could potentially be attributed to the exceptional chemical stability and faradaic reactions exhibited by NiO. The approximate value of the equivalent series resistance (R_s_) was ascertained using circuit fitting techniques, which was found to be 1.14 Ω. The straight slope parallel to the imaginary axis verifies the symmetric supercapacitor conventional capacitive behaviour. The y-axis of the phase angle is about 80° indicating ideal capacitive behavior. Also, capacitive behavior of the device can be measured the frequency at which the phase angle approaches 45° and found to be 2.2 Hz, showing outstanding capacitive nature with quick frequency response.

## Experimental

### Synthesis procedure

**Figure 5:**
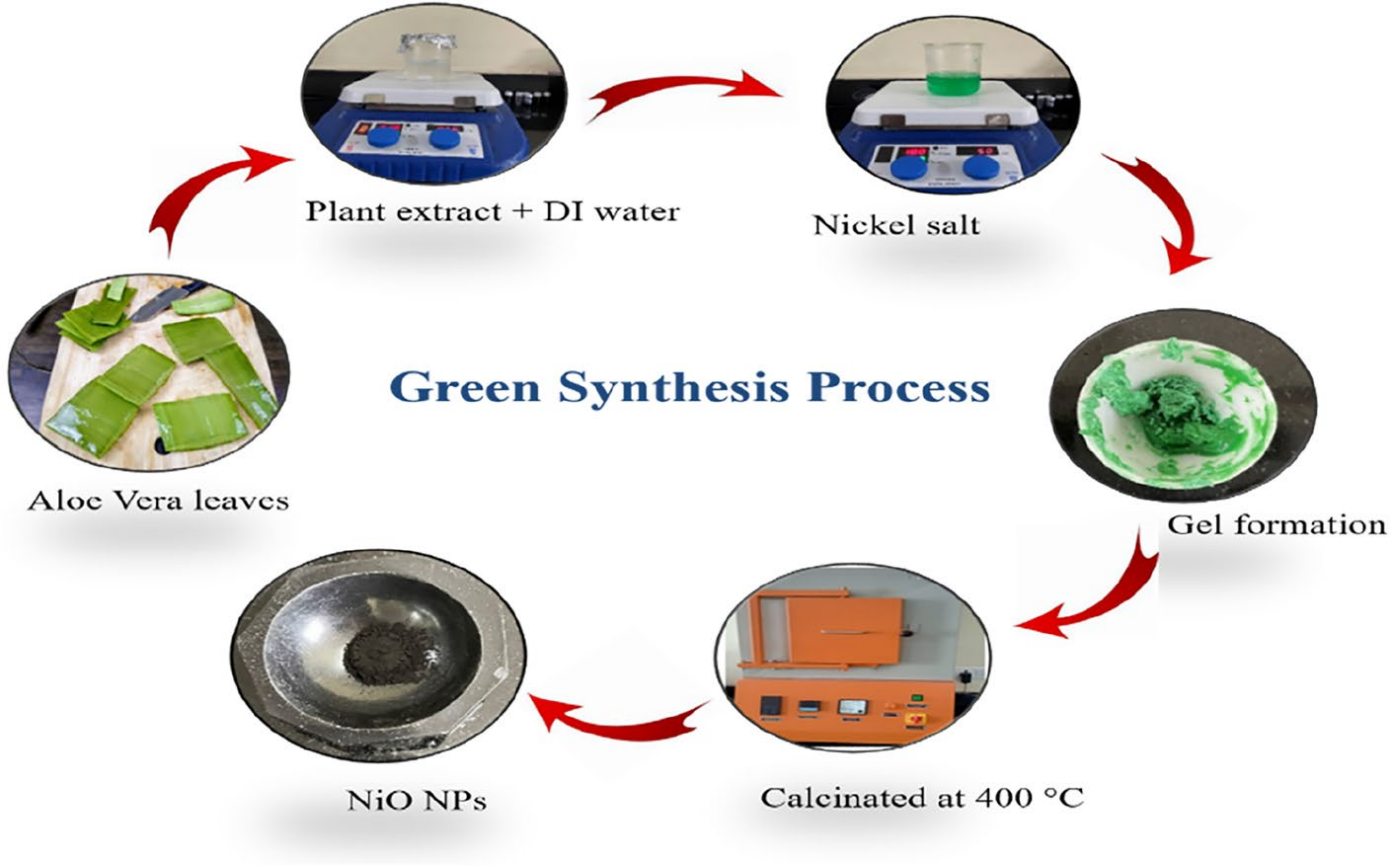
Schematic diagram of green synthesis process of NiO. NPs.

Pellets of KOH and nickel nitrate hexahydrate (Ni(NO_3_)_2_·6H_2_O) were obtained from Sigma Aldrich, which were used directly without any refinement. The aloe vera leaves were brought from a local market in Hisar, Haryana. The aloe vera leaves were taken and washed with distilled (DI) water, removed from the upper green layer and then cut into fine pieces. Finally, the extract was ground to form a colloidal suspension. Initially, 20 g of aloe vera colloidal suspension was added into 100 ml DI water and put into a magnetic stirrer at 75 °C for 1 h to form a transparent solution. After that, 10 g of Ni(NO_3_)_2_·6H_2_O was put into the transparent solution and boiled until it became a green paste. The consequent green gel was heated at 400 °C in a muffle furnace for 2 h in order to get NiO NPs (Eq. 7) as depicted in Fig. 5.

7 $${Ni({NO}_{3})}_{2} \xrightarrow{Aloe\, vera\, extract}{\to } Ni {(OH)}_{2} \xrightarrow{Calcined\, at\, 400^\circ\, C} NiO$$

### Material characterizations

The structural properties of synthesized material i.e., NiO NPs were studied using XRD, having monochromatic radiation with Cu K_α_ radiations varying 2θ from 5° to 90°. This diffractometer was used to calculate crystallite size and phase composition with known wavelength (λ = 1.54 Å) of X-ray using a powder sample. The FT-IR spectrum was recorded to find the vibrational modes present in the material using potassium bromide between wavenumber from 4000 to 400 cm^−1^. The morphology of the material was investigated using FE-SEM 1 µm and 100 nm at 15 kV accelerating voltage and TEM at 1µm and 100nm scales. A UV/vis spectrophotometer with a 200–800 nm wavelength range was used to evaluate the optical absorbance of the synthesized NPs after being dispersed in DI water. All the methods were carried out in accordance with relevant Institutional guidelines and regulations.

### Electrochemical measurements

All electrochemical analysis was done using CV, GCD and EIS in 2 M KOH as an electrolyte. Employing the potentiostat galvanostat (PGSTAT M204, Metrohm Autolab B.V., The Netherlands) within a three-electrode setup, electrochemical characteristics were investigated using the NOVA 2.0 software at ambient temperature. The reference electrode was a saturated Ag/AgCl, while a platinum wire was the auxiliary electrode. For the working electrode, a blend of active material (NiO NPs), activated charcoal and PVDF (binder) was formulated in an 8:1:1 ratio. To create a uniform slurry, a small quantity of N-methyl-2-pyrrolidinone (organic solvent) was added to the mixture, which was then stirred at room temperature for 24 h. Then prepared slurry was deposited into a pre-cleaned graphite sheet substrate (serves as a current collector) on a 1 × 1 cm^2^ area and vacuum oven dried for 24 h at 70 °C. The CV curve was observed at various scan rates (1, 5, 10, 20, 50 mV s^−1^) in a voltage window of 1V. The specific capacitance (C_s_) from the CV curves was calculated using the equation shown below^44^:

8 $${C}_{s}=\frac{1}{ms (Vf-Vi)}\underset{Vi}{\overset{Vf}{\int }}I\left(V\right) dv$$

where m denotes the deposited mass on the substrate, s is the scan rate, V_f_ and V_i_ are final and initial voltage and I(V) show the current response, respectively.

Furthermore, the charging and discharging were examined at different current densities (0.76, 1.53, 2.3, 3.9 and 6.2 A g^−1^) and the specific capacitance (C_s_) from GCD plots was calculated using the following equation^45^:

9 $${C}_{s}=\frac{I\times \Delta t}{m\times \Delta V}$$

where m denotes the mass deposited on the substrate, $\Delta V$ is voltage window and I show the current response, and $\Delta t$ is discharging time, respectively.

Subsequently, EIS was performed by varying frequency from 0.01 to 10^5^ Hz of the working electrode at an amplitude of 5 mV.

Then, the symmetric device was fabricated integrating anode and cathode with active material and electrodes were isolated using Whatman filter paper, which had been soaked in a 2 M KOH electrolyte and then sealed with insulating tape. An equal surface area (1 × 1 cm^2^) of active material was deposited on both electrodes.

The electrochemical behaviour of the symmetric device was also examined by CV, GCD and EIS measurements. To perform electrochemical analysis, a two-electrode setup was employed with each consisting of the same material NiO NPs with the same charge capacity. The CV curve was obtained at various scan rates viz. 5, 10, 20, 50,100, 150 and 200 mV s^−1^ with a voltage window of 1.2 V. The charging-discharging of the device was recorded at 1–10 A g^−1^ current density based on the mass loaded on the electrode. The specific capacitance (C_s_) was estimated by Eq. (10) and further energy density (E_S_) and power density (P_S_) was then determined from the C_s_ as given in Eqs. (11) and (12).

10 $${C}_{s}=\frac{2\times I\times \Delta t}{m\times \Delta V}$$

11 $${E}_{s}=\frac{Cs{\times \Delta V}^{2}}{2\times 3.6}$$

12 $${P}_{s}=\frac{{E}_{s}\times 3600 }{\Delta t}$$

where I is the charging/discharging current, Δt denotes the time of complete discharge, m represents mass deposited on both electrodes and ΔV signifies the voltage window.

## Conclusions

The successful synthesis of NiO NPs was achieved through an eco-friendly approach by utilizing a sustainable method involving Aloe vera plant extract as a reducing agent. The confirmation of NiO NP production was validated by XRD and FTIR spectra analyses. The resulting material exhibited remarkable electrochemical performance, having a significant specific capacitance of approximately 462 F g^−1^ at a scan rate of 1 mV s^−1^ and 336 F g^−1^ at a current density of 0.76 A g^−1^. Moreover, the application of NiO NPs in a symmetric device showed fascinating results such as a specific capacitance of 239 F g^−1^, a specific energy density of 47.8 Wh kg^−1^ and a power density of 545 W kg^−1^ within a 1.2 V voltage window. The device also maintained an excellent retention capacity of 89% after 2000 GCD cycles with 114% coulombic efficiency at a current of 10 A g^−1^. The novelty of this approach combines sustainable materials with the benefits principle of green synthesis, which involves utilizing simple, low-cost, low-temperature, environmentally friendly, economical and greener solvents. The NiO NPs that were produced have the potential to be employed as electrode materials in electrochemical energy storage systems due to the high density of faradic activities.

### Supplementary Information

Supplementary Information.

## Data Availability

The authors would like to confirm that all data generated or analyzed during this study are included in this published article.
