# Supplementary material for: Natural resource-derived NiO nanoparticles via aloe vera for high-performance symmetric supercapacitor
Source: Sci Rep. 2024 Mar 28;14:7389. doi: 10.1038/s41598-024-57606-w (PMC10978893; doi:10.1038/s41598-024-57606-w)
Supplement: Supplementary file 1 — Supplementary Information. [file 41598_2024_57606_MOESM1_ESM.docx]

**Supplementary Information**

**Electrochemical Performance of synthesized Nio as an electrode across various voltage windows:**

Cyclic voltammetry (CV) was performed on a single electrode within a three-electrode configuration. The reference and auxiliary electrodes consisted of Ag/AgCl and platinum wire, respectively, while the working electrode was composed of the synthesized active material, namely nickel oxide (NiO).

The CV was conducted across various voltage windows spanning from 0.0 V to 1.0 V, -0.2 V to 0.8 V, -0.4 V to 0.6 V, -0.6 V to 0.4 V, -0.8 V to 0.2 V, and -1.0 V to 0.0 V in Figure S1. The charge storage capacity is assessed based on the area under the curve, with a larger area indicating a higher specific capacitance. Noteworthy is that the potential window spanning from -1.0 to 0.0 demonstrates the largest area among all the windows. The specific capacitance values were determined using equation (8) mentioned in the manuscript, as 167 F g⁻¹, 212 F g⁻¹, 220 F g⁻¹, 276 F g⁻¹, 242 F g⁻¹, and 320 F g⁻¹ for the respective voltage windows of 0.0 V to 1.0 V, -0.2 V to 0.8 V, -0.4 V to 0.6 V, -0.6 V to 0.4 V, -0.8 V to 0.2 V and -1.0 V to 0.0 V as shown in Table S1.


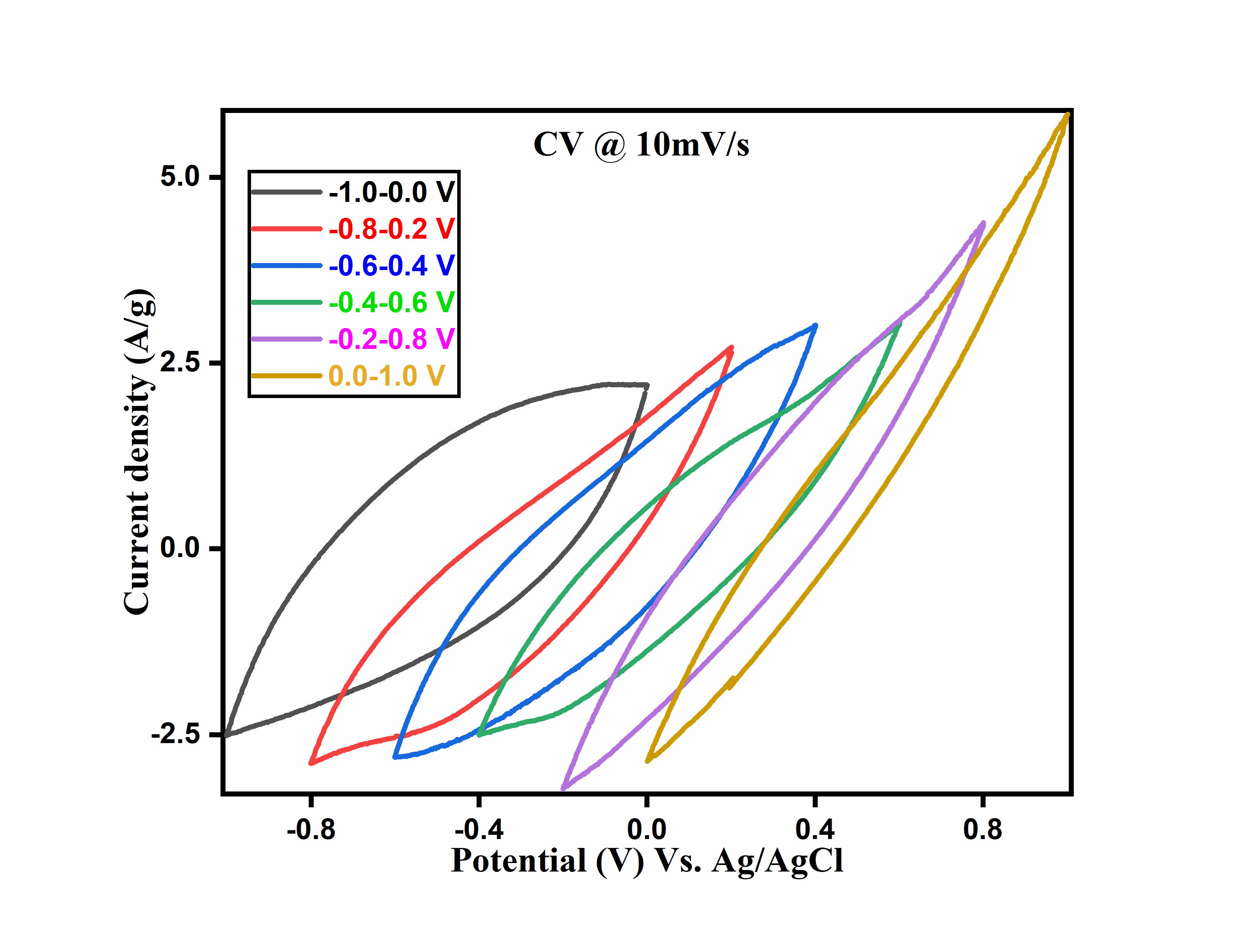


**Fig. S1** CV curve for NiO at different voltage windows at a scan rate of 10 mV/s.

**Table S1:** Specific capacitance at different voltage windows at 10 mV/s.

| **Potential window (V)** | -1 to 0 | -0.8 to 0.2 | -0.6 to 0.4 | -0.4 to 0.6 | -0.2 to 0.8 | 0 to 1 |
| --- | --- | --- | --- | --- | --- | --- |
| **Specific capacitance**  (F g⁻¹) | 320 | 242 | 276 | 220 | 212 | 167 |

Table S2 compiles research outcomes, depicting negative potential windows observed in investigations of NiO and diverse metal oxides. This concurs with the consistent observations documented in various research endeavors.

**Table S2:** Comparative analysis of various metal oxides exhibiting negative potential windows in the presence of KOH electrolyte.

| **S.N.** | **Material** | **Potential Window** | **Electrolyte** | **Specific Capacitance (F g⁻¹)** | **References** |
| --- | --- | --- | --- | --- | --- |
| **1.** | NiO | -1 to 0 | 2M KOH | 320 @ 10 mV/s | This work |
| **2.** | NiO | -0.4 to 1 | 1M KOH | 548 @ 1 mV/s | [[1](https://doi.org/10.1039/C4RA16776G) ] |
| **3.** | NiO Thin film | -1.05 to 0.75 | 2M KOH | 1000 @ 5 mV/s | [2] |
| **4.** | Mn-doped NiO | -0.5 to 0.3 | 6M KOH | 396.6 @ 0.5 A/g | [[3](https://doi.org/10.1080/21870764.2020.1793477)] |
| **5.** | MnO2 | -0.7 to 0.3 | 1 M KOH | 591.6 @ 5 mV/s | [[4](https://doi.org/10.1007/s10008-017-3557-8)] |
| **6.** | RuO2 | -0.8 to 0.4 | 6 M KOH | 584 | [[5](https://doi.org/10.1002/cjoc.200690212)] |
| **7.** | Fe2O3 | -1.2 to 0 | 6 M KOH | 218 @ 1 A/g | [6] |
| **8.** | CF-Fe2O3 | -1.15 to 0.2 | 2 M KOH | 1.56 F/cm^2^  F @ 10 mA/ cm^2^ | [[7](https://doi.org/10.1021/acs.jpcc.7b04330)] |

The charge storage mechanism within NiO potentially encompasses the intercalation and deintercalation of K+/OH- ions. In this intricate process, ions dynamically infiltrate and exit the active material, inducing reversible alterations in charge states. These transformations contribute significantly to energy storage within the electrode. Importantly, similar phenomena have been previously observed and reported in studies involving NiO and various other metal oxides [8, 9,10,11].

**References**

1. Jahromi, S. P., Pandikumar, A., Goh, B. T., Lim, Y. S., Basirun, W. J., Lim, H. N., & Huang, N. M. (2015). Influence of particle size on performance of a nickel oxide nanoparticle-based supercapacitor. Rsc Advances, 5(18), 14010-14019.
2. Kate, R. S., Khalate, S. A., & Deokate, R. J. (2017). Electrochemical properties of spray deposited nickel oxide (NiO) thin films for energy storage systems. *Journal of Analytical and Applied Pyrolysis*, *125*, 289-295.
3. Srikesh, G., & Nesaraj, A. S. (2020). Facile preparation and characterization of novel manganese-doped nickel oxide based nanostructured electrode materials for application in electrochemical supercapacitors. *Journal of Asian Ceramic Societies*, *8*(3), 835-847.
4. Ingole, S. M., Navale, S. T., Navale, Y. H., Dhole, I. A., Mane, R. S., Stadler, F. J., & Patil, V. B. (2017). Galvanostatically electroplated MnO 2 nanoplate-type electrode for potential electrochemical pseudocapacitor application. *Journal of Solid State Electrochemistry*, *21*, 1817-1826.
5. Wang, X. F., You, Z., & Ruan, D. B. (2006). A hybrid metal oxide supercapacitor in aqueous KOH electrolyte. *Chinese Journal of Chemistry*, *24*(9), 1126-1132.
6. Phakkhawan, A., Suksangrat, P., Srepusharawoot, P., Ruangchai, S., Klangtakai, P., Pimanpang, S., & Amornkitbamrung, V. (2022). Reagent-and solvent-mediated Fe2O3 morphologies and electrochemical mechanism of Fe2O3 supercapacitors. *Journal of Alloys and Compounds*, *919*, 165702.
7. Li, T., Yu, H., Zhi, L., Zhang, W., Dang, L., Liu, Z., & Lei, Z. (2017). Facile electrochemical fabrication of porous Fe2O3 nanosheets for flexible asymmetric supercapacitors. *The Journal of Physical Chemistry C*, *121*(35), 18982-18991.
8. Liu, X., Zhai, Z. Y., Chen, Z., Zhang, L. Z., Zhao, X. F., Si, F. Z., & Li, J. H. (2018). Engineering mesoporous NiO with enriched electrophilic Ni3+ and O− toward efficient oxygen evolution. *Catalysts*, *8*(8), 310. (OH-)
9. Liu, Y., Jiang, S. P., & Shao, Z. (2020). Intercalation pseudocapacitance in electrochemical energy storage: recent advances in fundamental understanding and materials development. *Materials Today Advances*, *7*, 100072. (cations)
10. Lu, J., Xu, W., Li, S., Liu, W., Javed, M. S., Liu, G., & Hu, C. (2018). Rational design of CuO nanostructures grown on carbon fiber fabrics with enhanced electrochemical performance for flexible supercapacitor. *Journal of Materials Science*, *53*, 739-748.
11. Ren, Z., Li, J., Ren, Y., Wang, S., Qiu, Y., & Yu, J. (2016). Large-scale synthesis of hybrid metal oxides through metal redox mechanism for high-performance pseudocapacitors. *Scientific Reports*, *6*(1), 20021.
